# Learning from Other Tumors: Pathways for Progress and Overcoming Challenges in Cholangiocarcinoma

**DOI:** 10.3390/cancers17010156

**Published:** 2025-01-06

**Authors:** Giulia Tesini, Chiara Braconi, Lorenza Rimassa, Rocio I. R. Macias

**Affiliations:** 1 Department of Biomedical Sciences, Humanitas University, Via Rita Levi Montalcini 4, Pieve Emanuele, 20072 Milan, Italy; lorenza.rimassa@hunimed.eu; 2 School of Cancer Sciences, University of Glasgow, Switchback Rd., Glasgow G61 1QH, UK; chiara.braconi@glasgow.ac.uk; 3 Beatson West of Scotland Cancer Centre, 1053 Great Western Rd., Glasgow G12 0YN, UK; 4 CRUK Scotland Cancer Centre, Glasgow G61 1BD, UK; 5 Medical Oncology and Hematology Unit, Humanitas Cancer Center, IRCCS Humanitas Research Hospital, Via A. Manzoni 56, Rozzano, 20089 Milan, Italy; 6 Experimental Hepatology and Drug Targeting (HEVEPHARM) Group, University of Salamanca, IBSAL, CIBERehd, Campus M. Unamuno s/n, 37007 Salamanca, Spain

## 1. Introduction

Cholangiocarcinoma (CCA) is a group of complex and heterogeneous tumors originating from the epithelial cells of bile ducts that can occur in intrahepatic, perihilar, or distal localizations. The global incidence of CCA varies geographically, with higher rates in Southeast Asia (>6 cases/100,000 inhabitants) compared to Western countries (0.5–4 cases/100,000 inhabitants), while its mortality rate is high due to late diagnosis and limited effective therapeutic options. It is estimated to be responsible for 2% of cancer-related deaths [1]. This tumor is gaining attention due to its increasing global incidence and mortality in recent decades, which has been observed especially in the intrahepatic CCA (iCCA) subtype [1,2]. The silent progression of this disease and the lack of identifiable risk factors, which hinder its detection in early stages of development, together with the high incidence of relapse biological aggressiveness, and primary and acquired resistance to pharmacological treatments [3,4], highlight the urgent need for further research and awareness campaigns to improve patient outcomes. Over the years, several cancers that were once considered untreatable have seen remarkable improvements in patient outcomes, largely due to advances in diagnostic techniques, detailed molecular profiling, innovative treatment strategies, and comprehensive patient support systems. While some of the valuable insights and lessons learned from these successful cases can be applied to CCA, this disease presents unique challenges that will require dedicated efforts to overcome.

## 2. Improving Early Detection and Diagnosis

Advances in early detection and diagnostic strategies have significantly improved outcomes in lung and colorectal cancers. Both tumors are good examples of where organized screening programs, biomarker discovery, and advances in imaging technologies have played a life-changing role. Low-dose computed tomography (LDCT) has become the gold standard for lung cancer screening and has demonstrated a substantial reduction in mortality by facilitating the detection of smaller localized tumors at stages suitable to curative interventions. Evidence from large-scale trials, such as the National Lung Screening Trial (NLST), showed a 20% relative reduction in lung cancer mortality among high-risk individuals screened with LDCT [5]. Similarly, colorectal cancer screening using non-invasive methods, such as fecal immunochemical tests (FIT) and endoscopic approaches, have revolutionized early detection practices. These tools not only identify premalignant polyps for removal but also improve survival rates by diagnosing cancers at earlier stages, when treatment is more effective. The success of colorectal cancer screening programs, as demonstrated in population-based studies, underscores the importance of maintaining adherence and accessibility to screening [6].

The establishment of effective early detection strategies for CCA is challenging due to its relatively low incidence, which renders mass screening programs less cost-effective. Symptoms such as jaundice, weight loss, and abdominal pain often do not appear until advanced stages, complicating early diagnosis. Biomarkers such as carbohydrate antigen 19-9 (CA19-9) are widely used, but their sensitivity and specificity are limited in the early stages of disease, and their accuracy is further compromised in the presence of benign biliary conditions [7]. Standard imaging techniques, such as computed tomography (CT) and magnetic resonance imaging (MRI), often have difficulty differentiating between malignant and benign lesions in the early stages of disease.

Some iCCAs are detected in patients with cirrhosis who are being monitored for the risk of developing HCC. Patients with primary sclerosing cholangitis (PSC), chronic hepatobiliary diseases, or a strong familial predisposition who are at high risk of developing CCA may be included in dedicated follow-up programs. However, the percentage of CCA patients with these underlying pathologies is small, and annual screening of patients with PSC with contrast-enhanced MRI and magnetic resonance cholangiopancreatography (MRCP) imaging, serum liver function tests, and CA19-9 proved ineffective in detecting cancer early enough to favor long-term survival [8].

Emerging techniques in circulating tumor DNA (ctDNA) analysis, used successfully in colorectal cancer [9], could be adapted for CCA. The number of studies investigating different types of biomarkers in serum or bile for the prediction of CCA development prior to clinical evidence of malignancy in patients with PSC and other hepatobiliary diseases is high [10], but the results need to be validated in international cohorts before being implemented in the clinic.

## 3. Expanding Molecular Profiling

Liquid biopsy has become part of the therapeutic algorithm of breast and lung cancer, contributing to the optimization of treatment sequencing through longitudinal assessments of mutations of interest. For instance, for patients with estrogen receptor-positive HER2-negative metastatic breast cancer, guidelines from the American Society of Clinical Oncology recommend that estrogen receptor 1 mutations are investigated at the time of progression to endocrine therapy, preferably by using ctDNA analysis [11]. Likewise, in non-squamous non-small cell lung cancer (NSCLC) with epidermal growth factor receptor (*EGFR*) alterations, liquid biopsy to detect *EGFR* T790M mutations upon progression to first- or second-generation anti-EGFR tyrosine kinase inhibitors (TKIs) was indicated to assess eligibility to osimertinib, before the latter became the standard of care in first-line treatment [12,13].

While genomic profiling at baseline is already part of the management of patients with CCA, longitudinal assessments of key molecular alterations have not been sufficiently explored. However, liquid biopsy for patients with CCA harboring therapeutically targetable alterations could be considered. In particular, patients with fibroblast growth factor receptor 2 (*FGFR2*) fusions or rearrangements could be ideal candidates, as multiple FGFR2 inhibitors are available, although it should be noted that access to genomic assays and novel drugs varies significantly across countries. Both pemigatinib and futibatinib have now entered clinical practice [14,15,16], and other anti-FGFR agents (e.g., tinengotinib) may be accessed within clinical trials [17]. Futibatinib is more active than pemigatinib against point mutations in the gatekeeper and molecular brake residues, partly because it is an irreversible FGFR1-4 inhibitor [18]. Moreover, novel findings suggest that *RAS* mutations, *FGFR2* gatekeeper mutations, and polyclonality are more frequently observed upon progression to futibatinib, providing preliminary evidence that irreversible FGFR inhibitors can induce a distinct pattern of acquired resistance mechanisms compared to reversible ones that can be detected and therapeutically targeted [19].

ctDNA analysis before the beginning of targeted therapy could help to select the most optimal FGFR inhibitor, and longitudinal assessments during treatment could allow for the prompt detection of secondary resistance mechanisms, leading to the early introduction of new drugs and the optimization of the sequencing of different available agents.

## 4. Immunotherapy and Combination Strategies

Immune-checkpoint inhibitors (ICIs) have improved outcomes across multiple solid tumors. Notably, in non-squamous NSCLC, pembrolizumab in combination with chemotherapy more than doubled median overall survival (OS) compared to chemotherapy alone, while in squamous NSCLC, it led to a less marked yet still significant improvement [20,21,22,23]. In both cases, 5-year OS rates were approximately 20% [21,23]. By contrast, in patients with CCA, first-line therapy with ICIs in combination with cisplatin and gemcitabine yielded 2- and 3-year OS rates of 15–20% [24,25,26,27]. Nonetheless, these rates are twice those achieved with chemotherapy alone; consequently, the identification of factors predictive of response to immunotherapy is a topic of interest.

Traditional predictive biomarkers of response to immunotherapy include programmed death-ligand 1 (PD-L1) expression, tumor mutational burden (TMB), and microsatellite instability (MSI). However, PD-L1 expression, which is used to assess eligibility to single-agent ICIs in NSCLC [28,29], does not correlate to response to chemoimmunotherapy in CCA [24,26]. Likewise, TMB-high does not predict response to ICIs in CCA, while it is a known predictive factor in many other solid tumors, such as endometrial and anal cancer [30]. MSI status is an exception, but only 1–2% of CCAs are MSI-high [31]. Genomic alterations do not influence response to chemoimmunotherapy among patients with CCA. In NSCLC, EGFR mutations are associated with low response rates to ICIs and an increased risk of developing immune-related adverse events [32]. However, a translational analysis of the TOPAZ-1 trial of cisplatin, gemcitabine, and durvalumab in first-line treatment has shown similar efficacy outcomes between patients with or without therapeutically targetable alterations [33].

Therefore, current research is focusing on expanding the use of ICIs and finding ways to overcome treatment resistance. Combinatorial strategies with agents directed against vascular endothelial growth factor (VEGF), such as bevacizumab, have proved effective in other tumors, as anti-angiogenic drugs can increase responses to PD-L1 inhibitors by inducing the maturation of dendritic cells and a reduction in the activity of immunosuppressive cells [34]. In patients with hepatocellular carcinoma (HCC), the combination of bevacizumab and atezolizumab yielded a 6-month survival improvement compared to sorafenib, becoming a standard option for advanced disease [35,36]. Similarly, lenvatinib, a multitargeted TKI with anti-VEGF receptor activity, has demonstrated efficacy in combination with pembrolizumab and transarterial chemoembolization in patients with intermediate stage HCC [37]. In CCA, both the IMbrave151 phase 2 study of cisplatin and gemcitabine in association with atezolizumab and bevacizumab/placebo in first-line treatment and the LEAP-005 phase 2 trial of lenvatinib and pembrolizumab for pretreated patients showed preliminary efficacy signals [38,39]. However, these results need to be confirmed in large randomized phase 3 trials.

Lastly, a broader landscape of immune checkpoints has recently emerged. For instance, the presence of T cell immunoreceptors with immunoglobulin and immunoreceptor tyro-sine-based inhibitory motif domains (TIGIT) is a negative prognostic factor in non-squamous NSCLC [40]. While targeting TIGIT alone yielded unsatisfactory results, trials including the dual inhibition of TIGIT and PD-L1 using bispecific antibodies or combination treatments are ongoing in several types of tumors, including CCA [40]. The ARTEMIDE-Biliary-01 trial (NCT06109779) of adjuvant chemotherapy in association with rilvegostomig (an anti-TIGIT and anti-PD-1 bispecific antibody) or placebo will also help shed light on the role of ICIs in curative settings. Similarly, the GEMINI trial (NCT05775159) of cisplatin and gemcitabine in association with rilvegostomig or volrustomig (an anti-PD-1 and anti-cytotoxic T lymphocyte-associated protein 4 bispecific antibody) will contribute to expanding our knowledge on the role of ICIs in advanced settings.

## 5. Building Support Systems

Patient support and advocacy networks have transformed the landscape of hematological malignancies and breast cancer care. These organizations provide critical services: (i) informing patients and caregivers about their diseases and available treatments; (ii) supporting innovative research to improve diagnostic tools and therapies; and (iii) influencing health policy to ensure equitable access to treatments and address disparities in care delivery. The success of the Susan G. Komen Foundation for breast cancer and the Leukemia & Lymphoma Society for hematological malignancies in raising awareness, funds, and improving care outcomes is a model for other cancers.

Despite being a rare tumor, some organizations, such as the AMMF in the United Kingdom or the Cholangiocarcinoma Foundation in the United States, have managed to raise awareness of CCA and, although there is still a long way to go, this has encouraged new associations to be created more recently in other countries such as Italy, France, and Spain. Expanding CCA-specific patient advocacy groups is essential to raise awareness among patients, healthcare professionals, and policymakers, as well as to influence research priorities and funding allocation and facilitate access to clinical trials and innovative treatments.

The joint work of these organizations and scientific networks, such as ENSCCA (European Network for the Study of Cholangiocarcinoma) and ICRN (International Cholangiocarcinoma Research Network), provides frameworks for interdisciplinary research and collaboration. These collaborations have been strengthened by the European Union funding for scientific and technological research networks, known as European Cooperation in Science and Technology (COST) actions, which promote the connection of researchers and innovators across Europe and beyond through the recently funded EUROCHOLANGIONET and Precision-BTC-Network projects that focus on biliary cancer.

CCA can no longer remain an overlooked malignancy. By learning from advancements in other cancers and fostering interdisciplinary teamwork, we can address the pressing challenges of late diagnosis, limited treatment, and unequal access to care. The integration of innovative diagnostics, precision medicine, and immunotherapy, supported by patient advocacy and international collaboration, has the potential to transform CCA outcomes. Together, we can pave the way for a future where CCA patients receive equitable, effective, and timely care worldwide.
